# Enhancing the Wound Healing Effect of Conditioned Medium Collected from Mesenchymal Stem Cells with High Passage Number Using Bioreducible Nanoparticles

**DOI:** 10.3390/ijms20194835

**Published:** 2019-09-28

**Authors:** Gwang-Bum Im, Yeong Hwan Kim, Yu-Jin Kim, Sung-Won Kim, Euiyoung Jung, Gun-Jae Jeong, Ke Wang, Jinheung Kim, Dong-Ik Kim, Tae-Hyung Kim, Gi-Ra Yi, Taekyung Yu, Suk Ho Bhang

**Affiliations:** 1 School of Chemical Engineering, Sungkyunkwan University, Suwon 16419, Korea; lki1005@skku.edu (G.-B.I.); iva9110@gmail.com (Y.H.K.); yujinkim1003@gmail.com (Y.-J.K.); tjdnjdl90@skku.edu (S.-W.K.); kewang@hust.edu.cn (K.W.); yigira@skku.edu (G.-R.Y.); 2 Department of Chemical Engineering, Kyung Hee University, Youngin 17104, Korea; jey9207@khu.ac.kr; 3 Department of Chemistry and Nano Science, Ewha Womans University, Seoul 120-750, Korea; jinheung@ewha.ac.kr; 4 Division of Vascular Surgery, Samsung Medical Center, Sungkyunkwan University School of Medicine, Seoul 06351, Korea; jgj814@skku.edu (G.-J.J.); dikim@skku.edu (D.-I.K.); 5 School of Integrative Engineering, Chung-Ang University, Seoul 06974, Korea; thkim0512@cau.ac.kr

**Keywords:** angiogenesis, high passage, mesenchymal stem cell, nanoparticle

## Abstract

Injecting human mesenchymal stem cells (hMSCs) at wound sites is known to have a therapeutic effect; however, hMSCs have several limitations, such as low viability and poor engraftment after injection, as well as a potential risk of oncogenesis. The use of a conditioned medium (CM) was suggested as an alternative method for treating various wounds instead of direct hMSC administration. In addition to not having the adverse effects associated with hMSCs, a CM can be easily mass produced and can be stored for long-term, thereby making it useful for clinical applications. In general, a CM is collected from hMSCs with low passage number; whereas, the hMSCs with high passage number are usually discarded because of their low therapeutic efficacy as a result of reduced angiogenic factor secretion. Herein, we used a CM collected from high passage number (passage 12, P12) hMSCs treated with gold-iron nanoparticles (AuFe NPs). Our AuFe NPs were designed to release the iron ion intracellularly via endocytosis. Endosomes with low pH can dissolve iron from AuFe NPs, and thus, the intracellularly released iron ions up-regulate the hypoxia-inducible factor 1α and vascular endothelial growth factor (VEGF) expression. Through this mechanism, AuFe NPs improve the amount of VEGF expression from P12 hMSCs so that it is comparable to the amount of VEGF expression from low passage number (passage 6, P6), without treatment. Furthermore, we injected the CM retrieved from P12 MSCs treated with AuFe NPs in the mouse skin wound model (AuFe P12 group). AuFe P12 group revealed significantly enhanced angiogenesis in the mouse skin wound model compared to the high passage hMSC CM-injected group. Moreover, the result from the AuFe P12 group was similar to that of the low passage hMSC CM-injected group. Both the AuFe P12 group and low passage hMSC CM-injected group presented significantly enhanced re-epithelization, angiogenesis, and tissue remodeling compared to the high passage hMSC CM-injected group. This study reveals a new strategy for tissue regeneration based on CM injection without considering the high cell passage count.

## 1. Introduction

Human mesenchymal stem cells (hMSCs) are known to have wound healing effects. Previous studies reported that hMSCs can accelerate the proliferation of human dermal fibroblast cells via several processes such as secretion of paracrine factors, which promote re-epithelialization and angiogenesis in cutaneous wounds [1,2,3]. Paracrine factors secreted from the transplanted hMSCs spread to the dermis and epidermis through diffusion and thus accelerate wound healing [4]. Among the various paracrine factors secreted from hMSC, vascular endothelial growth factor (VEGF) is crucial in wound healing [5,6]. Although hMSCs have therapeutic effects, the viability of MSCs decreases after transplantation in wound site with hypoxic condition [7,8]. In particular, harsh environments, such as hypoxic conditions in wound sites, trigger the death stimuli to transplanted stem cells [9]. As most stem cells transplanted into the damaged tissue undergo rapid cell death (apoptosis and necrosis), it is necessary to inject numerous stem cells in the damaged tissue as a therapeutic measure. This requirement of mass preparation increases the time and cost of the treatment, which is a major concern [10]. Moreover, administrating stem cells in the damaged tissue itself is associated with potential risk of carcinogenesis [11,12].

Injecting the conditioned medium (CM) collected from stem cells can be an alternative method for stem cell injection-based therapies. CM retrieved from hMSC culture contains various paracrine factors that can stimulate wound healing [13,14,15]. The CM does not contain stem cells—which can potentially form tumors—and is also not associated with carcinogenesis. However, it was reported that the concentration of therapeutic paracrine factors in CM retrieved from conventional 2D cell culture without any treatment was insufficient to induce appropriate tissue repair and regeneration [14]. Moreover, the adult stem cells lose their stemness and capacity to secret paracrine factors as they proliferate continuously for a long period [16,17]. Due to the reduction in paracrine factor secretion in adult stem cells with a high passage number, CM-based therapies are predominantly based on adult stem cells with low passage number [18,19,20,21]. Although sufficient CM and stem cells can be collected after a few passages, most of the stem cells with high passage number are considered to be biowaste and are discarded due to their low therapeutic efficacy.

In this study, gold-iron nanoparticles (AuFe NPs) were employed to improve the secretion of paracrine factors from adult stem cells by controlling the cellular microenvironment via Fe ion delivery. Previous studies demonstrated that intracellularly delivered Fe ion can up-regulate the secretion of paracrine factors in adult stem cells [22,23]. Moreover, it was discovered that the expression of specific genes such as CXCR4 and hypoxia-inducible factor 1α (HIF-1α), which enhance secretion of angiogenic paracrine factors, can be increased by the Fe nanoparticles [23]. It is well known that HIF-1α expression leads to VEGF gene expression from hMSCs [24]. Our novel AuFe NPs were designed to release the Fe ion under low pH conditions, which mimic the low pH conditions of the endosomes (pH 4.5–5.5). Nanoparticles are easily engulfed by cells via endocytosis [25]. AuFe NPs which undergo endocytosis can be trapped within the endosomes where they would be exposed to low pH conditions, which are ideal for Fe ion release via AuFe NPs degradation. It was conjectured that if Fe ions can be successfully delivered into hMSCs with high passage number using pH-sensitive AuFe NPs, hMSCs with high passage number might be able to secrete more paracrine factors compared to those without any treatment. As a result, a large amount of CM with high concentration of paracrine factors can be collected from high passage number hMSCs.

In this experiment, the viability of hMSCs was measured after treatment with various amounts of AuFe NPs to determine the optimized concentration of AuFe required for enhancing the secretion of the angiogenic paracrine factors, without resulting in any cytotoxic effects. The cell viability tests were performed via the cell viability kit (CCK-8) analysis and fluorescein diacetate and ethidium bromide (FDA/EB) assay. Using reverse transcriptase polymerase chain reaction (RT-PCR) and quantitative real-time polymerase chain reaction (qRT-PCR), VEGF expression from high passage number (P12) hMSCs treated with AuFe NPs was compared with that of high passage number hMSCs without NP treatment and low passage number hMSCs (P6). After treatment of hMSCs with the optimized concentration of AuFe NPs, CM was retrieved from each group, for further analyses. To confirm the therapeutic efficacy of CM obtained from AuFe NP-treated high passage number hMSCs, a mouse skin wound closing model was injected with these CM. CM collected from high passage number hMSCs without AuFe NP treatment served as another experimental group, whereas CM collected from hMSCs with low passage number served as the positive control group. We tested whether the CM collected from AuFe NP-treated hMSCs promoted the regeneration of the skin wound by examining angiogenesis, re-epithelization, and tissue remodeling, based on morphological changes in wound site histology, and based on the results of immunohistochemistry and qRT-PCR.

## 2. Results

### 2.1. Characterization of pH-Sensitive AuFe NPs

**Figure 1:**
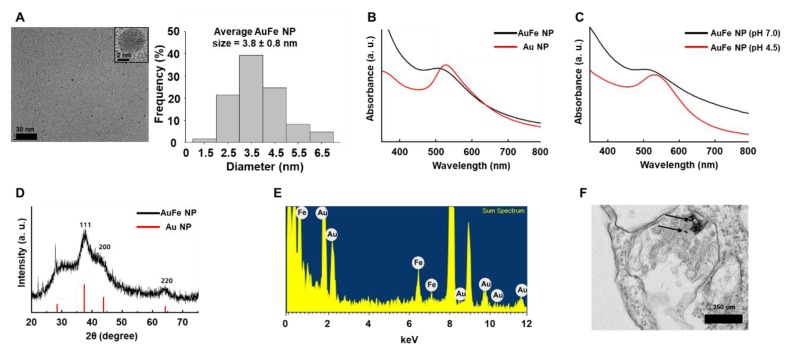
(**A**) Transmission electron microscopy (TEM) image of AuFe NPs (left) with size distribution (right). UV-Vis spectra of AuFe NPs with Au NPs at (**B**) neutral pH 7.0 and (**C**) pH 4.5. Black line indicates AuFe NP and red line indicates Au NP. (**D**) Powder X-ray diffraction (XRD) patterns of AuFe NPs and Au NPs. (**E**) Energy-dispersive X-ray spectroscopy (EDS) spectrum of AuFe NPs. (**F**) TEM image of an hMSC with AuFe NPs (black arrows: AuFe NPs in hMSC, scale bar represents 250 μm).

Our novel pH-sensitive AuFe NPs were round in shape with average size (3.8 ± 0.8 nm), as indicated by transmission electron microscopy (TEM) images in Figure 1A. As depicted in Figure 1B, AuFe NPs at pH 7.0 exhibited a plasmon resonance peak at 520 nm, whereas Au NPs exhibited a peak at 530 nm. At low pH, Fe ions were released from AuFe NPs and a change in the peak was detected (Figure 1C). Figure 1D depicts the X-ray diffraction (XRD) patterns in which AuFe NPs moved to slightly higher angles compared to Au NPs. The Energy-dispersive X-ray spectroscopy (EDS) spectrum revealed that the AuFe NPs comprised only Au and Fe, with no other elements (Figure 1E). Figure 1F illustrates the intracellular uptake of AuFe NPs by hMSCs, 1 h after AuFe NP treatment, as indicated by the black arrows.

### 2.2. Optimization of AuFe NP Concentration for the Treatment of hMSCs, Based on Cytotoxicity and VEGF Gene Expression

**Figure 2:**
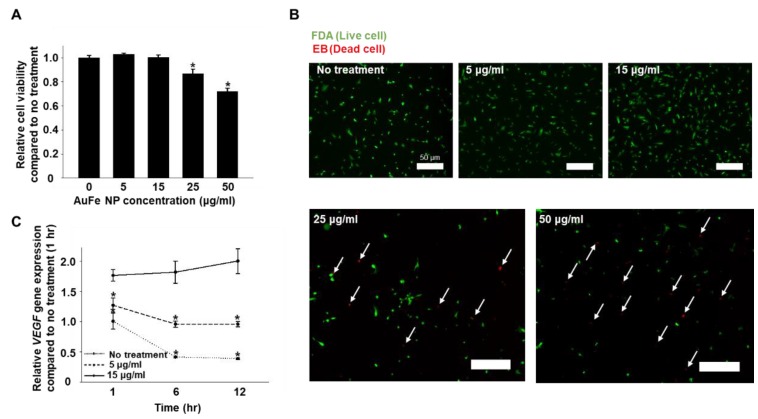
(**A**) Cytotoxicity test performed on hMSCs, 24 h after treatment with various concentrations of AuFe NP (0, 5, 15, 25, and 50 μg/mL) (*n* = 4, * *p* < 0.05 compared to no treatment). (**B**) FDA/EB assay to measure hMSC viability, 24 h after treatment with various concentrations of AuFe NP. Live cells are stained in green and dead cells (white arrows) are stained in red. Scale bars represent 50 μm. (**C**) Relative mRNA expression of VEGF in hMSCs treated with various concentrations of AuFe NP for 1, 6, and 12 h, as evaluated by qRT-PCR (*n* = 4, * *p* < 0.05 versus AuFe NPs 15 μg/mL group).

The cytotoxicity of AuFe NPs was evaluated on Day 1 after the treatment of hMSCs with various concentrations of AuFe NPs (0, 5, 15, 25, and 50 μg/mL). AuFe NPs exerted no cytotoxic effect on hMSCs at concentrations below 15 μg/mL. The FDA/EB assay revealed increased dead cell count after AuFe NP treatment when the concentration was higher than 15 μg/mL (25 and 50 μg/mL). Based on the cell viability test, low concentrations of AuFe NPs (0, 5, and 15 μg/mL) were selected for use in further experiments. As depicted in Figure 2C, hMSCs treated with 15 μg/mL of AuFe NP exhibited the highest expression of VEGF compared to the other groups. Furthermore, VEGF expression at 1 h after AuFe NP treatment revealed no significant difference with respect to 6 and 12 h post treatment. hMSCs treated with 5 μg/mL of AuFe NP exhibited enhanced VEGF expression compared to hMSCs without NP treatment. At 6 and 12 h, hMSCs without treatment as well as those treated with 5 μg/mL of AuFe NP exhibited reduced VEGF expression with respect to that at the corresponding 1 h timepoint. In this study, AuFe NPs were internalized into the intracellular endosome of hMSCs via endocytosis. Thereafter, the iron ions released from endosome enhanced the VEGF gene expression of hMSCs (Figure 2C).

### 2.3. Effects of AuFe NPs on hMSCs In Vitro

**Figure 3:**
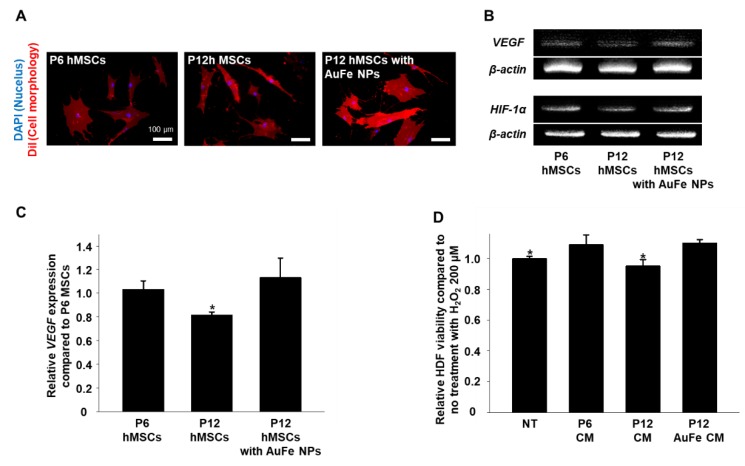
(**A**) DiI staining (blue: nucleus, red: cellular membrane) of hMSCs with low passage (P6) without NP treatment, hMSCs with high passage (P12) without AuFe NP treatment, and hMSCs with high passage (P12) with AuFe NP treatment. (**B**) VEGF and HIF-1α expression in P6 hMSCs without NP treatment and P12 hMSCs without or with AuFe NP treatment, as evaluated by RT-PCR. (**C**) Relative VEGF expression in P12 hMSCs without or with AuFe NP treatment, with respect to that in P6 hMSCs without NP treatment (*n* = 4, * *p* < 0.05 versus P12 hMSCs with AuFe NPs group). (**D**) Relative cell viability of human dermal fibroblasts (HDFs) cultured using CM collected from NT; Fresh medium, P6 CM; P6 hMSCs without NP treatment, P12 CM; P12 hMSCs without NP treatment, and P12 AuFe CM; P12 hMSCs with NP treatment under hypoxic cinditions (H_2_O_2_, 200 μM) (*n* = 4, * *p* < 0.05 versus P12 AuFe CM group).

As the number of passages increases, the morphology of the hMSCs becomes more bipolar and VEGF expression decreases compared to that in the low passage number hMSCs [26,27]. Figure 3A shows that high passage number hMSCs (P12) were bipolar and more elongated as compared to the low passage number hMSCs (P6). Indeed, the cell morphology revealed no specific difference between P12 hMSCs with and without AuFe NP treatment. Compared to P12 hMSCs without AuFe NP treatment (Figure 3B,C), AuFe NP-treated P12 hMSCs exhibited significantly increased VEGF expression. Interestingly, a similar fold increase in VEGF expression in AuFe NP-treated P12 hMSCs was observed relative to that in P6 hMSCs. Also, we observed enhancement of HIF-1α expression from P12 hMSCs treated with AuFe NPs compared to P12 hMSCs (Figure 3B). Previous studies reported that high passage number cells show decrement of HIF-1α expression compared to low passage number cells under normoxic condition [28,29,30,31]. The CM collected from P6 hMSCs without NP treatment and P12 hMSCs with AuFe NP treatment prevented cell death in human dermal fibroblasts (HDF) cultured under hypoxic conditions which mimic the in vivo environment prior to in vivo mouse skin wound site (H_2_O_2_, 200 μM, Dulbecco’s modified Eagle’s medium (DMEM) without serum) [32]. Previous studies repeated that VEGF can inhibit apoptosis in stem cells under hypoxic conditions [33,34].

### 2.4. Therapeutic Efficacy of CM Collected from hMSCs with AuFe NP Treatment

**Figure 4:**
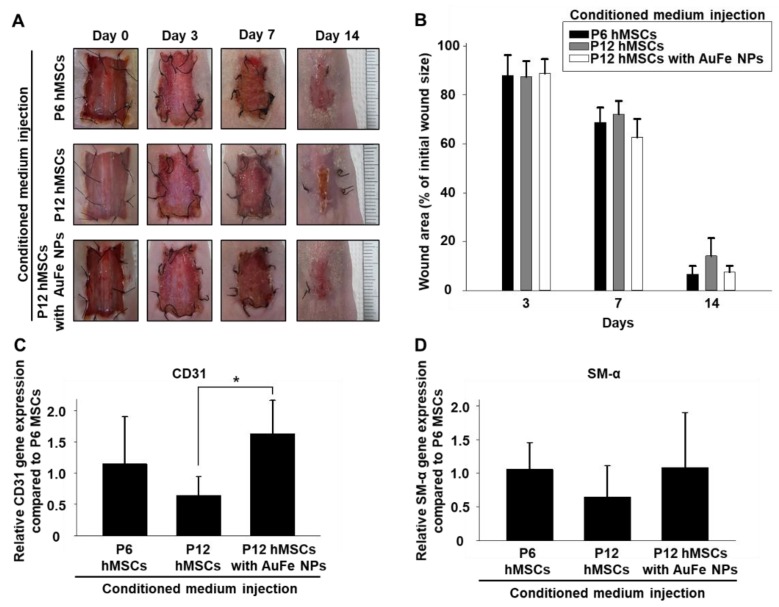
(**A**) Representative images of full-thickness skin wounds at various time points after injecting CM collected from the untreated P6 (P6 NT group) and P12 hMSCs (P12 NT group), as well as AuFe NP-treated P12 hMSCs (P12 AuFe group). (**B**) Wound closure rates (*n* = 4). Relative gene expression of (**C**) CD31 and (**D**) SM α-actin in wound regions (*n* = 4, * *p* < 0.05) at 14 days after treatment.

The CM collected from cultures of P6 hMSCs, P12 hMSCs, and AuFe NP-treated P12 hMSCs, were injected into mice models of skin wounds. Figure 4C depicts that AuFe NP-treated P12 hMSCs exhibit significantly increased CD31 expression compared to the P12 hMSCs without treatment; however, no significant difference was observed among various cell groups with respect to wound closing at 14 days after the treatment (Figure 4A,B). As illustrated in Figure 4D, SM-α expression did not exhibit any significant difference among various cell groups.

### 2.5. Improved Wound Healing Induced by CM Collected from AuFe NP-Treated hMSCs

**Figure 5:**
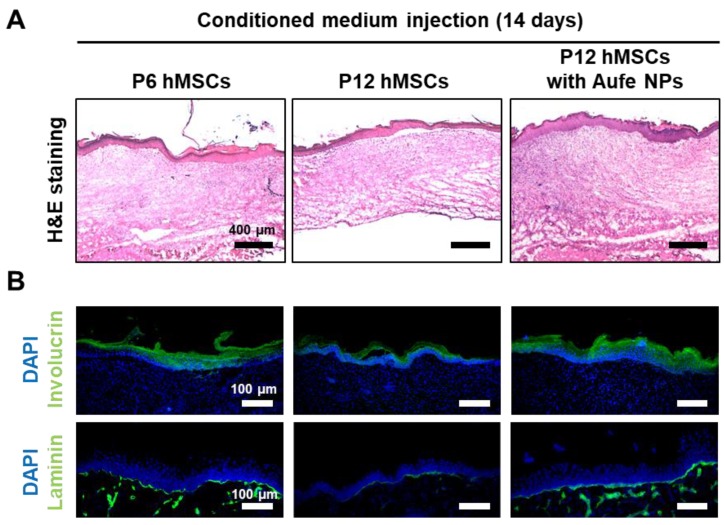
(**A**) Hematoxylin and eosin-stained sections of the mid-portion of the wound at 14 days after treatment. Scale bars represent 400 µm. (**B**) Immunohistochemical staining of involucrin (green) in the epidermis and laminin (green) in the basal layer along with 4′,6-diamidino-2-phenylindole (DAPI) (blue) staining in the healing wound at 14 days after treatment. Scale bars represent 100 µm.

Unique tissue layers, which can be found in the normal skin tissue were partially detected in CM collected from P6 hMSCs and AuFe NP-treated P12 hMSCs (Figure 5A). Due to the presence of involucrin and laminin positive signals at the wound sites, regeneration of the epidermis and basal layers in P6 hMSCs and AuFe NP-treated P12 hMSCs were relatively enhanced compared to those of P12 hMSCs group [35]. According to the *in vivo* data (qRT-PCR, wound closing ratio, and tissue staining), the appearance of wound sites revealed no significant difference at 14 days after the treatment among the three groups; however, the relative amount of involucrin and laminin positive signals increased in P6 hMSCs and AuFe NP-treated P12 hMSC compared to P12 hMSCs without treatment, which indicates improved internal tissue regeneration in the wound sites compared to P12 hMSCs without treatment.

## 3. Discussion

Although CM can be used as an alternative source for stem cell therapy, the therapies based on CM are limited, as stem cells with low passage number can secrete larger amounts of therapeutic paracrine factors, which are required for tissue regeneration, as compared to stem cells with high passage number [27]. Angiogenic growth factors such as VEGF play a pivotal role in the wound healing process when present in sufficient amounts. Previous studies reported that transplanting hMSCs into cutaneous wound sites can considerably improve tissue regeneration through paracrine factor secretion [1,2,3]. Because direct stem cell transplantation into the wound site is associated with a potential risk of tumor formation and low cell viability along with poor engraftment posttransplantation due to unfavorable microenvironments such as hypoxia, CM might be a good alternative approach for tissue regeneration based on the stem cell transplantation; however, low concentration of several growth factors in the CM limits its therapeutic efficacy compared to that of stem cell transplantation [36]. Previously, researchers used huge cell culture systems to collect a lot of CM from stem cells [37]. When stem cells proliferate continuously for a longer period, the concentration of paracrine factors secreted from the stem cells decrease extensively [17]. Thus, stem cells with a high passage number are usually regarded as a biowaste and are discarded. These cells were excluded from cell transplantation due to the loss of stemness and original morphology [17,27]. As CM injection only includes the growth factors secreted from the stem cells, increasing growth factor secretion from high passage number stem cells might be useful in skin wound closure. To preserve the appropriate amounts of paracrine factors secreted from stem cells regardless of the passage number, we used AuFe NPs to treat high passage number stem cells.

Previous studies reported that the expression of HIF-1α can increase when Fe ions enter the stem cells [22,23]. HIF-1α binds to the VEGF promoter and acts as a transcription activator of VEGF [24]; Based on the difference in degradation between Au and Fe, Fe ions was efficiently delivered into the cytoplasm without causing abrupt cell death (Figure 2A,B). AuFe NPs were exposed to low pH (4–5) conditions via endocytosis. However, in contrast to Fe, Au does not degrade at pH 4–5. Therefore, at low pH, Fe gets degraded and is released into the cytoplasm as Fe ions, whereas Au remains as a nanoparticle. Thus, using AuFe NP caused no cell damage, and the Fe ions introduced into the cells could enhance the secretion of paracrine factors from high passage number hMSCs. To determine the possibility of using AuFe NP for advanced CM collection in order to aid the closure of skin wounds, we performed experiments for optimizing the amount of AuFe NPs required for treating hMSCs and enhancing the secretion of paracrine factors following treatment.

AuFe NP-treated hMSCs presented significantly enhanced VEGF expression and exhibited therapeutic effects on the skin wounds. Our AuFe NPs were synthesized using an eco-friendly method that employed water, and not an organic solvent; hence, they can be easily employed in a clinical setting. Moreover, AuFe NPs are approximately 3.8 nm in diameter, which can be accumulated to a lesser degree in the liver and spleen (compared to large sized nanoparticles) (Figure 1A) [38]. As illustrated in Figure 2, extensive AuFe NP treatment causes cell death, whereas lower concentrations of AuFe NP up-regulate VEGF expression. In this study, we found that treating hMSCs with optimized concentrations of AuFe NPs could lead to increased VEGF expression and enhanced cell viability under hypoxic conditions. Similar to the previous findings [39], Fe ions released from AuFe NPs after endocytosis up-regulated HIF-1α expression (Figure 3B). The enhanced VEGF expression in AuFe NP-treated high passage number hMSCs suggests that the CM collected from these hMSCs might have advanced angiogenic and antiapoptotic effects when injected into the wound sites as compared to those of the conventional CM [33,40]. Figure 3D illustrated the increase in HDF viability under hypoxic conditions after treatment with CM collected from P6 hMSCs and AuFe NP-treated P12 hMSCs. Previous research showed that fibroblasts play important roles in the wound healing process [41,42,43]. Through mimicking the hypoxic condition of the skin wound environment *in vitro* before the *in vivo* test, the result showing the viability and proliferation of fibroblasts could provide the possibility of improved therapeutic effect induced by CMs. Appearance of wound closure in the mouse model with CM derived from with NP-treated P12 hMSCs indicated no significant difference at 14 days after the treatment compared to those of other groups (Figure 4A,B). However, as depicted in Figure 4C,D, angiogenesis was significantly increased up on injecting CM collected from AuFe NP-treated P12 hMSCs compared to that from P12 hMSCs. Expression of CD31 revealed that angiogenesis after injecting CM derived from AuFe NP-treated P12 hMSCs was significantly enhanced in comparison to the conventional CM method (P6 NT group). In addition to angiogenesis, re-epithelization also presented similar results. Collectively, our novel pH-sensitive AuFe NPs could successfully enhance the wound healing ability of high passage number hMSCs.

This research demonstrated the effect of using AuFe NPs and the availability of high passage number adult stem cells in wound healing using the nanoparticles. Treatment of high passage number hMSCs with AuFe NPs significantly enhanced the secretion of angiogenic paracrine factors and the expression of antiapoptotic genes compared to that in high passage number hMSCs without AuFe NPs treatment. Accordingly, the CM collected from AuFe NP-treated high passage number hMSCs dramatically enhanced the wound healing in a mouse skin wound model compared to the CM collected from high passage number hMSCs without nanoparticle treatment. Thus, based on our results, it is possible to collect large volumes of therapeutically effective CM derived from hMSCs regardless of their passage number. Further investigation regarding up-regulation of other growth factor types that would lead to enhanced therapeutic efficacy, is under progress. Our result implies that AuFe NPs treatment of high passage number stem cells could suggest a new platform for collecting therapeutically efficient CM.

## 4. Materials and Methods

### 4.1. Materials

Gold (III) chloride hydrate (HAuCl_4_∙xH_2_O, 99.995%), iron (III) chloride (FeCl_3_, 98%), poly(vinyl pyrrolidone) (PVP, *M*_w_ = 55,000 Da), sodium borohydride (NaBH_4_), hydrochloric acid (HCl, 37%), and sodium hydroxide (NaOH, 9%) were purchased from Sigma-Aldrich (St. Louis, MO, USA), and were used without further purification.

### 4.2. Synthesis of AuFe NPs

To synthesize AuFe NPs, 9 mL of an aqueous solution containing 100 mg PVP was prepared at room temperature (RT; of (20–24) °C) with magnetic stirring at 600 rpm for 15 min. About 1 mL NaBH_4_ (4 mg) solution was injected into the PVP solution. Thereafter, 1 mL HAuCl_4_∙xH_2_O (4 mg) solution and 1 mL FeCl_3_ (2 mg) solution were added to the reacting solution, and were allowed to age at the same temperature for 15 min. The final product was collected via centrifugation, and washed several times with deionized (DI)-water and acetone. The AuFe NPs were well redispersed in the DI-water.

### 4.3. Characterization

TEM images and EDS spectrum were captured using a field emission electron microscope (JEM-2100F, JEOL, Tokyo, Japan) operating at 200 kV. XRD patterns were obtained by X-ray diffractometer (D-MAX/A, Rigaku, Tokyo, Japan) at 35 kV and 35 mA. The UV-Vis spectra were recorded using a Jasco UV-Vis spectrophotometer (Cary 60 UV-vis, Agilent Technologies, Santa Clara, CA, USA) within the range of 250–850 nm.

### 4.4. Fe Ion Release from the AuFe NPs under Acidic Condition

The amount of Fe ion released from the AuFe NPs at different pH was determined using UV-Vis spectroscopy (Cary 60 UV-vis, Agilent Technologies). The pH 4.5 was considered to be the microenvironment of endosome, and pH 7 was considered to be a neutral condition of cell culture medium. HCl was added to the phosphate buffered saline (PBS, Gibco BRL, Gaithersburg, MD, USA) solution to adjust two different pH conditions of pH 4.5 and 7. The AuFe NPs were then dispersed in PBS with different pHs of pH 4.5 and 7 at RT. After 12 h, each sample was centrifuged, to separate the NPs from the PBS.

### 4.5. Cell Culture

The MSCs derived from human were purchased from Lonza (Basel, Switzerland), and were cultured in Dulbecco’s modified Eagle’s medium (DMEM, Gibco BRL), supplemented with 10% (*v/v*) fetal bovine serum (Gibco BRL) and 1% (*v/v*) penicillin/streptomycin (Gibco BRL). The cells were incubated at 37 °C with 5% CO_2_ saturation. The medium was changed every 2 days. In this study, hMSCs in passage 6 and passage 12 were used. To collect CM from the hMSCs treated with or without AuFe NPs for the *in vivo* experiment, hMSCs were treated with AuFe NPs (15 μg/mL, 1 h) and washed three times with PBS. Then, serum free cell culture media were supplied to the hMSCs. Forty-eight hours after culturing the hMSCs with serum free medium, CMs were retrieved and injected to mouse wound closing models.

### 4.6. Measurement of Cytotoxicity of AuFe NPs

Cell viability was evaluated using a cell counting kit-8 (CCK-8, Dojindo Molecular Technologies, Inc., Kumamoto, Japan). The CCK-8 assay measures the amount of formazan dye that is reduced by the intracellular dehydrogenase activities. The number of living cells is proportional to the amount of formazan dye. In general, the hMSCs (1 × 10^4^ cells/well with 400 μL serum free medium) in passage 12 were cultured on 24 well plates with various concentrations of AuFe NPs for 24 h, and were rinsed with PBS thrice. After replenishing the wells with fresh medium, CCK-8 solution was added into each well, and the cells were incubated for 2 h. Furthermore, the absorbance was measured at 450 nm using a plate reader (Infinite F50, Tecan, Männedorf, Switzerland). The cell viability was calculated as the percentage of viable cells relative to the AuFe NPs-untreated cells (*n* = 4 per group). HDFs (3 × 10^4^ cells/well with 400 μL conditioned medium collected from various group) were treated with H_2_O_2_ 200 μM solution. Then, living cells were measured by CCK-8 assay at 12 h after treatment. Cellular membranes and cell adhesion were evaluated by 1,1′-dioctadecyl-3,3,3′,3′-tetramethylindocarbocyanine perchlorate (DiI, Sigma-Aldrich) staining. After treating the passage 12 hMSCs with AuFe NPs for 1 h, the cells were treated with the DiI solution (6.25 µM) and incubated for 30 min at 37 °C. The cells were then washed twice in PBS. Cells were fixed with paraformaldehyde 4% solution for 10 min, and were then washed in PBS. After DAPI (Vector Laboratories, Burlingame, CA, USA) staining, DiI fluorescence was measured by a fluorescence microscope (IX71, Olympus, Tokyo, Japan). We evaluated the cell size and length using Photoshop CC program (Adobe Systems, San Jose, CA, USA) [44]. Live/Dead assays were performed with fluorescein diacetate (FDA; sigma) and ethidium bromide (EB; sigma). FDA (green) stains the cytoplasm of viable cells, whereas EB (red) stains the nuclei of nonviable cells. The staining solution was freshly prepared by mixing 10 mL of FDA stock solution (1.5 mg/mL of FDA in dimethyl sulfoxide), 5 mL of EB stock solution (1 mg/mL of EB in PBS), and 3 mL of PBS. Then, the staining solution was applied to the cells and incubated for 3–5 min at 37 °C. After staining, the samples were washed twice with PBS and examined using a fluorescence microscope (DFC 3000 G, Leica, Wetzlar, Germany).

### 4.7. qRT-PCR

qRT-PCR was used to quantify the relative gene expression levels of vascular endothelial growth factor VEGF, and micro blood vessel marker CD31 (platelet endothelial cell adhesion molecule) and SM-α (one of the earliest markers of mural cell (vascular smooth muscle cells and pericytes) development in vertebrates to stabilize or provide contractility to blood vessels). Total ribonucleic acid (RNA) was extracted from samples (10^5^ cells per each sample) using 1 mL TRIzol reagent (Life Technologies, Inc., Carlsbad, CA, USA) and 200 μL chloroform. The lysed samples were centrifuged at 12,000 rpm for 10 min at 4 °C. The RNA pellets were washed with 75% (*v/v*) ethanol in water, and were then dried. After drying, the samples were dissolved in RNase-free water. For qRT-PCR, the SsoAdvanced™ Universal SYBR Green Supermix kit (Bio-Rad, Hercules, CA, USA) and the CFX Connect™ real-time PCR detection system (Bio-Rad), were used according to the manufacturer’s instruction. Table 1 lists the primers used for qRT-PCR. We used human gene primers to evaluate the enhancement of human specific VEGF gene expression from hMSCs (*in vitro*) induced by AuFe NPs. Mouse gene primers were used to evaluate the mouse specific CD31 and SM-α gene expression from wound site (*in vivo*) after CM injection.

### 4.8. RT-PCR

The 10^5^ of hMSCs in presence or absence of AuFe NP treatment for 1 h were lysed in TRIzol reagent. Total RNA was extracted and precipitated with 80% (*v/v*) solution of isopropanol in water, and the RNA pellets were washed with 75% (*v/v*) solution of ethanol in water, air-dried, and dissolved in 0.1% (*v/v*) diethyl pyrocarbonate-treated water. Reverse transcription was performed using 10 μL of 2× Easy Taq SuperMix (TransGen Biotechnology, Beijing, China), 0.5 μL of cDNA, 0.5 μL of each primer, and 8.5 μL of sterile pure H_2_O, followed by PCR amplification of the synthesized complementary deoxyribonucleic acid. PCR comprised 35 cycles of denaturing (94 °C, 30 s), annealing (58 °C, 45 s), and extension (72 °C, 45 s), with a final extension at 72 °C for 10 min. PCR was followed by electrophoresis on a 2% (*w/v*) agarose gel, and visualization by ethidium bromide staining. PCR products were analyzed using a gel documentation system (WGD-30, Daihan Scientific, Korea). The β-actin served as an internal control. Table 2 lists the primers used for RT-PCR.

### 4.9. Intracellular Distribution of AuFe NPs

The hMSCs were cultured on a 150 mm dish and were incubated with 15 μg/mL AuFe for 1 h (1 × 10^6^ cells/dish, activated with 15 μg/mL of AuFe NPs and supplied with 20 mL of serum free medium). The cells were then fixed using Karnovsky’s fixative for 4 h at 4 °C, and were rinsed thrice with cold 0.05 M cacodylate buffer. The cells were fixed with 1% osmium tetraoxide for 2 h at 4 °C, and were then washed twice with cold distilled water. The samples were treated with 0.5% uranyl acetate overnight at 4 °C, dehydrated using graded ethanol concentrations of 30, 50, 70, 80, 90, 95, and 100%, rinsed with propylene oxide, and were eventually embedded in Spurr’s resin, which was then polymerized at 70 °C for 24 h. Thin sections of 100 nm thickness were obtained using an ultramicrotome (Leica, Wetzlar, German), collected on 200 mesh copper grids, and observed by TEM (JEM-1010, JEOL, Tokyo, Japan).

### 4.10. Wound Treatment

Six-week-old female athymic mice (20 g of body weight, Orient Bio, Inc., Sungnam, Korea) were intraperitoneally anesthetized with xylazine (10 mg/kg) and ketamine (100 mg/kg). A square shaped full-thickness wound (2.0 × 2.0 cm^2^) was created on the back of each mouse. Mice with skin wounds were randomly divided into three experimental groups. The wounds were treated with CM collected from hMSCs with low passage number (P6 NT group, *n* = 4), CM collected from hMSCs with high passage number without AuFe NP treatment (P12 NT group, *n* = 4). CM collected from hMSCs with high passage number with AuFe NP treatment (P12 AuFe group, *n* = 4). 2 mL of CMs according to the group were collected under identical cell culture conditions (10^5^ of hMSCs/well, cultured with serum free medium for 2 days). To prevent the wound by contracture, 8 sutures were placed at the border of the wound with 6-0 sutures (Ethicon, Somerville, NJ, USA), and the wound margins were anchored to the underlying muscle fascia. Daily injection of CM (200 μL/wound) was continuously performed for 4 days. After cell injection, the wounds were covered with gauze. Wound healing statuses were followed up to 14 days after the treatment. The films covering the wounds were changed at 0, 3, 7, 10, 14 days to capture the macroscopic wound images. All animals received care according to the guidelines for the care and use of laboratory animals of Sungkyunkwan university (Approved number: SKKUIACUC2017-05-03-3, September 2018).

### 4.11. In Vivo Wound Closing Ratio

The macroscopic wound area was quantified by processing the images captured at various time points by tracing the wound margin, and calculating the pixel area relating it to the ruler, using a fine-resolution computer mouse. The location of the advancing margin of wound closure was defined as the grossly visible margin of epithelial migration toward the center of the wound, and over the granulation tissue bed. The wound area was calculated as the percentage of the initial wound area ([wound area at time]/[initial wound area] × 100%). Morphometric analysis was performed on digital images using the imaging software (Photoshop CC, Adobe Systems).

### 4.12. Histology

Microscopic tissue regeneration was observed by H&E-stained tissue sections using a light microscope (CKX53, Olympus, Tokyo, Japan). Skin tissue samples were fixed in formaldehyde, dehydrated with a concentration of 20% sucrose, and embedded in optimum cutting temperature (OCT) compound (SciGen Scientific, Gardenas, CA, USA). Specimens were sliced into 10 µm thick sections, and were stained with H&E staining, in order to examine tissue regeneration.

### 4.13. Immunohistochemistry

For immunohistochemical staining, the samples embedded in OCT compound were cut into 10 μm thick sections at −22 °C. To stain the microvessels, immunohistochemistry was performed on sections with involucrin (Abcam), and laminin (Abcam) antibodies. involucrin^+^, and laminin^+^ signals were visualized with fluorescein-isothiocyanate-conjugated secondary antibodies (Jackson Immuno Research Laboratories, West Grove, PA, USA). The sections were counterstained with DAPI, and examined by fluorescence microscopy (IX71, Olympus, Tokyo, Japan).

### 4.14. Statistical Analysis

All quantitative data were expressed as the mean ± standard deviation. Statistical analysis was performed by analysis of variance (ANOVA) using a Bonferroni test. A *p* value < 0.05 was considered to be statistically significant.

## Figures and Tables

**Table 1:** Sequences for primers used in qRT-PCR.

Primer	Sequence
Human GAPDH	F: 5′-GTC GGA GTC AAC GGA TTT GG-3′ R: 5′-GGG TGG AAT CA TTG GAA CAT-3′
Human VEGF	F: 5′-GAG GGC AGA ATC ATC ACG AAG T-3′ R: 5′-CAC CAG GGT CTC GAT TGG AT-3′
Mouse CD31	F: 5′-CAA ACA GAA ACC CGT GGA GAT G-3′ R: 5′-ACC GTA ATG GCT GTT GGC TTC-3′
Mouse SM-α	F: 5′-CAG GCA TGG ATG GCA TCA ATC AC-3′ R: 5′-ACT CTA GCT GTG AAG TCA GTG TCG-3′

**Table 2:** Sequences for primers used in RT-PCR.

Primer	Sequence
Human β-actin	F: 5′-GCA CTC TTC CAG CCT TCC TTC C-3′ R: 5′-TCA CCT TCA CCG TTC CAG TTT TT-3′
Human VEGF	F: 5′-GCA GAA GGA GGA GGG CAG AAT-3′ R: 5′-ACA CTC CAG GCC CTC GTC ATT-3′
Human HIF-1α	F: 5′-TAT GAC CTG CTT GGT GCT GA-3′ R: 5′-GGG AGA AAA TCA AGT CGT GC-3′
